# Steroid hormones and human choriogonadotropin influence the distribution of alpha6-integrin and desmoplakin 1 in gland-like endometrial epithelial spheroids

**DOI:** 10.1007/s00418-020-01960-z

**Published:** 2021-01-27

**Authors:** V. U. Buck, M. T. Kohlen, A. K. Sternberg, B. Rösing, J. Neulen, R. E. Leube, I. Classen-Linke

**Affiliations:** 1 Institute of Molecular and Cellular Anatomy, Uniklinik RWTH Aachen University, Wendlingweg 2, 52074 Aachen, Germany; 2 Clinic for Gynaecological Endocrinology and Reproductive Medicine, Uniklinik RWTH Aachen University, Pauwelsstraße 30, 52074 Aachen, Germany

**Keywords:** Human endometrium, Ishikawa cell line, 3D cell culture system, Epithelial polarity, Endometrial receptivity, Cell adhesion

## Abstract

In human glandular endometrial epithelial cells, desmosomal and adherens junction proteins have been shown to extend from a subapically restricted lateral position to the entire lateral membrane during the implantation window of the menstrual cycle. Similarly, a menstrual cycle stage-dependent redistribution of the extracellular matrix adhesion protein α6-integrin has been reported. These changes are believed to be important for endometrial receptiveness and successful embryo implantation. To prove the hypothesis that steroid hormones and human choriogonadotropin can induce the redistribution of these adhesion molecules, we used the human endometrial cell line Ishikawa in a 3D culture system. Gland-like spheroids were grown in reconstituted basement membrane (Matrigel™). The lumen-bearing spheroids were treated for 2 or 4 days with ovarian steroids or human choriogonadotropin and then assessed by immunofluorescence microscopy. In addition, human endometrial biopsies were obtained from patients, who were in therapy for assisted reproductive technology, and were examined in parallel. Lateral redistribution of the desmosomal plaque protein desmoplakin 1 was observed in the spheroids treated either with progesterone, medroxyprogesterone acetate or human choriogonadotropin. Furthermore, the extracellular matrix adhesion protein α6-integrin showed an increased lateral membrane localization upon gestagen stimulation in the 3D culture system. The results of this study demonstrate that the 3D endometrial Ishikawa cell culture might be suited as an experimental model system to prove the effect of hormonal changes like those occurring during the window of implantation.

**Supplementary Information:**

The online version contains supplementary material available at 10.1007/s00418-020-01960-z.

## Introduction

Implantation of the human embryo is a crucial step for the initiation of human pregnancy (Norwitz et al. 2001). This initial step is rate limiting for the success of pregnancy. Studies on assisted reproductive technology (ART) suggest that only a third of all embryos succeed in implanting into the endometrium (Kasius et al. 2014; Koot et al. 2012; Macklon et al. 2002). Due to ethical restrictions to study early human embryo implantation, clinical data are based on estimates. The best way to address questions about early embryo-maternal dialogue and trophoblast-endometrial interaction in a vital 3D context is therefore the use of appropriate cell culture systems.

In the context of basic reproductive sciences, the invasion of maternal uterine endometrium by trophectoderm-derived cells provides insight into fundamental aspects of benign, non-cancerous tissue infiltration. In contrast to other tissues, as for example oviduct or peritoneum, the receptivity of the endometrial mucosa is highly selective for the implanting blastocyst. The endometrial receptivity is achieved by altered differentiation of the participating tissue elements and by cellular cross talk involving fibroblasts, immune cells, endothelial cells and epithelial cells (Sharkey and Macklon 2013). The change of EECs into the receptive state is reflected by a reduction of cell polarity (Denker 1993, 1994; Thie et al. 1996; Whitby et al. 2017). Our previous observations in clinical specimens, that were obtained from women of reproductive age, suggest that a change in EEC polarity in vivo is caused by changing ovarian steroid hormone levels (Buck et al. 2012). Apico-basal redistribution of lateral junctional complexes was observed in this study in EEC glands during the course of the menstrual cycle. Other studies showed that these glands are invaded by extravillous trophoblast cells (EVT) during early implantation, a mechanism that is assumed to provide the early embryo with glandular secretion products for nourishment (Burton et al. 2002; Moser et al. 2010). Using a 3D confrontation culture system, we showed that a decrease in EEC polarity leads to an increased invasiveness of EVT cells (Buck et al. 2015). The three well-established EEC lines HEC-1-A (highly polarized), Ishikawa (moderately polarized) and RL95-2 cells (poorly polarized) (Hannan et al. 2010) were compared in that study. For the present study on hormonal regulation of EEC polarity, the Ishikawa cell line was selected as it represents an intermediate state of EEC polarity which is most likely present prior to the switch from non-receptive to receptive endometrium during the implantation window of the menstrual cycle. To mimic clinical treatments, which primarily stimulate endometrial differentiation of ART patients with 17β-estradiol (E2) and progesterone (P4), we supplemented 3D cultures of gland-like Ishikawa spheroids with these hormones. In addition, we applied the synthetic progestin medroxyprogesterone acetate (MPA) which is widely used in clinical treatments. MPA has also been frequently used in cell culture systems because of its longer half-life compared to P4, which is metabolized in vitro within 18 h (Ghatge et al. 2005). To account for a novel strategy in the ART field, namely trying to increase endometrial receptivity by intrauterine application of human choriogonadotropin (hCG), we also tested for a possible hCG effect on EEC polarity in our system.

The current study was set up to clarify whether the redistribution of cell adhesion proteins is influenced directly by administration of ovarian steroid hormones or by hCG in vitro. This would provide another mechanistic puzzle piece to our working hypothesis, that a reduction of human EEC polarity is regulated by steroid hormones to make the epithelial cells more permissive for implantation and further placentation.

## Materials and methods

### Cell culture

Ishikawa cells (ECACC 99040201, RRID: CVCL_2529) were maintained at 37 °C and 5% CO_2_ in culture medium (1:1 Dulbecco's modified Eagle’s medium:Ham’s F12 phenol red free; C.C.Pro, Oberdorla, Germany) containing 10% steroid hormone-free fetal calf serum (C.C.Pro) supplemented with 2 mM l-glutamine (Gibco, Paisley, UK) and 1% PSF (100 U/ml penicillin, 100 μg/ml streptomycin, 25 μg/ml fungizone; PAA, Pasching, Austria). Cells were split twice a week.

### 3D in vitro model

Ishikawa cells were grown up to 90% confluence in T25 cell culture flasks (Greiner Bio-One, Frickenhausen, Germany), washed with phosphate buffered saline (PBS) containing 0.02% (w/v) 2,2′,2″,2‴-(ethane-1,2-diyldinitrilo-)tetraacetic acid (EDTA; Sigma-Aldrich, Saint-Louis, MO, USA) for 5 min at 37 °C and incubated in PBS containing 0.25% (w/v) trypsin (BD, Sparks, MD, USA) and 0.02% EDTA for 1 min. Remaining cell clusters were dissociated by 20 times up and down pipetting using a 1000 μl pipet tip. The resulting single cell suspension was transferred into serum-free growth medium and mixed with an equal volume of ice-cold, growth factor-reduced Matrigel™ (BD Biosciences, Bedford, MA, USA) at a final density of 5 × 10^5^ cells/ml. 20 μl droplets of the Matrigel™-cell suspension were allowed to solidify for 45 min at 37 °C and 5% CO_2_. Cells were cultured in growth medium containing fetal calf serum with a medium change every second day. Beginning on day 4, hormones were added for 2 or 4 days. Final concentrations were 1 × 10^–8^ M for 17β-estradiol (E2; Sigma-Aldrich), 1 × 10^–6^ M for progesterone (P4; Sigma-Aldrich), 1 × 10^–6^ M for medroxyprogesterone acetate (MPA; Sigma-Aldrich), and 50 IU per ml cell culture medium for human choriogonadotropin (hCG; Ferring Pharmaceuticals, Kiel, Germany). As a control, the hormonal diluent ethanol was added to the medium (v/v 0.03%). Samples for evaluation were taken on day 2 or 4 after stimulation.

### Human biopsies

Endometrial biopsies were obtained from 11 31–40 year-old women, who were undergoing ART. Previous to an actual embryo transfer, patients underwent a simulation cycle for diagnostic purposes. In this simulation cycle, the patients were first treated with estrogen to promote endometrial growth. When an appropriate endometrial height was confirmed by sonographic control, 2 × 200 mg progesterone per day were vaginally applied for 6 days. Tissue samples were then taken with a pipelle catheter (Gynétics Medical products, Lommel, Belgium) at the Clinic for Gynaecological Endocrinology and Reproductive Medicine of the Uniklinik RWTH Aachen University. The use of the tissues was approved by the Ethics Committee of the Medical Faculty of the University of Aachen (EK 201/14 and EK 074/16). Fresh biopsies were fixed in 3.7% formalin and embedded in paraffin. Dating of the biopsies (Table 1) was performed on 5 µm thick sections according to the Noyes criteria (Noyes et al. 1950) and by immunohistochemical detection of estrogen and progesterone receptors and the proliferation marker Ki-67 as described in more detail by (Alfer et al. 2020).

**Table 1:** Biopsies of ART patients

Patient-code	Age	Therapy	Histological dating according to Noyes et al. (1950)
763	34	WOI-Cycle	17
440	36	WOI-Cycle	17–19
767	40	WOI-Cycle	18–19
466	36	WOI-Cycle	18–19
467	37	WOI-Cycle	18–19
266	36	WOI-Cycle	19
757	33	WOI-Cycle	19
736	31	WOI-Cycle	20
748	31	WOI-Cycle	20–21
503	34	WOI-Cycle	21
739	31	WOI-Cycle	22

### Immunohistochemistry

Detailed information on all antibodies and controls is provided in Table 2.

**Table 2:** List of antibodies and sera for immunohistochemistry

	Antigen/Protein	Species/Type/Isotype	Clone/Catalog #	Lot #	Source	Dilution % (v/v)
*Primary antibodies*	$\alpha$6-Integrin/CD49f	Rat, monoclonal IgG2a	GoH3/MAB13501	INW0117051	Beckman Coulter, Marseille, France	1:100
	$\alpha$6-Integrin	Rabbit, monoclonal, IgG	EPR18124/ab181551	GR3174340-6, GR3174340-8	Abcam, Cambridge, UK	1:200
	Actin	Rabbit, polyclonal	A2066	018M4753V	Sigma-Aldrich, Saint-Louis, MO, USA	1:5,000
	$\upbeta$4-Integrin/CD104	Rat, monoclonal, IgG2b $\upkappa$	439-9B/555719	45416, 6132805, 7117773	BD Pharmingen, Erembodegem, Belgium	1:200
	Desmoplakin 1	Guinea pig, polyclonal	(DP-1)	508281, 6101311, 706111	Progen Biotechnik, Heidelberg, Germany	1:500
	Ki-67	Rabbit, monoclonal, IgG	SP6/RBK027-05	P973, U391	Zytomed Systems, Berlin, Germany	1:200
	Zonula occludens 1	Rabbit, polyclonal	40-2200	1100434A, RB231622	Invitrogen, Camarillo, CA, USA	1:200
*Secondary antibodies*	Alexa Fluor 488 anti-rabbit	Goat F${(\mathrm{ab})}_{2}$	A-11070	1705868, 1907301	Invitrogen, Eugene, OR, USA	1:1000
	Alexa Fluor 488 anti-mouse	Goat	A-11029	1423008	Invitrogen, Eugene, OR, USA	1:500
	Alexa Fluor 555 anti-guinea pig	Goat	A21435	1666303	Invitrogen, Eugene, OR, USA	1:1000
	Alexa Fluor 555 anti-rat	Goat	A-21434	737676, 1846286	Invitrogen, Eugene, OR, USA	1:1000
*Control*	Rabbit negative control	Rabbit, polyclonal, IgG	AB-105-C	ER1416031	R&D Systems, Abingdon, UK	
	Mouse negative control	Mouse, monoclonal, IgG1	X0931	20032923	Dako, Glostrup, Denmark	
	Rat negative control	Rat, monoclonal, IgG2a	16-4321-81	E06650-1630	eBioscience, Waltham, MA, USA	
	Rat negative control	Rat, monoclonal, IgG2b	559478	29985	BD Pharmingen, Franklin Lakes, NJ, USA	

Ishikawa monolayers were washed twice with PBS at room temperature and fixed for 10 min in 3.7% formaldehyde in PBS. After washing in PBS, the cells were treated with 0.5% Triton X-100 in PBS for 5 min and washed afterwards in distilled water.

Endometrial spheroids on coverslips were washed once with PBS at room temperature. Cells were then fixed in methanol at − 20 °C for 5 min, air dried for 15 min and rehydrated in PBS for another 15 min at room temperature.

For immunofluorescence staining, antibodies were diluted in PBS supplemented with 1.5% bovine serum albumin. After incubation of cells with primary antibodies over night at room temperature, cells were washed three times for 45 min with PBS at room temperature. For nuclear staining 1 μg/ml Hoechst 33342 (Sigma-Aldrich) was added to the secondary antibody solution and incubated in the dark over night at room temperature. Coverslips were washed again three times for 45 min and rinsed briefly with deionized water before mounting on a 50 μl droplet of 30 °C warm Mowiol (Sigma-Aldrich) on a glass slide and stored at 4 °C until microscopic assessment. For double immunolabelling, both primary antibodies were applied simultaneously, as were the secondary antibodies.

Immunohistology of biopsies was performed on 5 μm thick microtome sections on standard glass slides. Sections were dewaxed in a descending alcohol series and rehydrated in PBS. Antigen retrieval was performed for 4 × 5 min in the microwave at 600 W in citrate buffer. Antibody reaction was carried out using the ZytoChem-Plus HRP Polymer Kit (Zytomed-Systems, Berlin, Germany). Incubation of the primary antibodies was carried out over night at 4 °C, secondary antibodies for 30 min at room temperature. Antigens were visualized by AEC (3-Amino-9-ethylcarbazol) and sections were counterstained by incubation in Mayer’s Hematoxylin solution (Merck, Darmstadt, Germany) for 90 s followed by treatment with tap water for 10 min. The sections were then mounted in glycerol gelatine (Merck, Darmstadt, Germany).

### Microscopy and image processing

Cell culture images were taken with PrimoVert (Zeiss MicroImaging, Jena, Germany) and a 10 × objective lens. Transmitted light images of the paraffin sections were recorded with an Axiophot (Zeiss) and a 40 × objective lens using the Zeiss AxioCam ICc 5 (12-bit, 2452 × 2056 pixel) and ZEN software (Zeiss, RRID: SCR_013672). Transmitted light images of spheroids in Matrigel™ were recorded in an Axio Imager M.2 microscope (Zeiss) with a 40 × objective lens using color camera ICc1 (Zeiss) and AxioVision software (Zeiss, RRID: SCR_002677). Differential interference contrast (DIC) and epifluorescence images were recorded using a Zeiss Axiocam MRm microscope (12-bit, 1388 × 1040 pixel) that was equipped with an ApoTome.2 unit. Fluorescence image processing was performed using the ImageJ-based program FIJI (https://imagej.nih.gov/ij/, RRID: SCR_002285). Cell culture images were also processed with Fiji and converted to an 8-bit format.

## Results

Detailed information on the number of experiments and assessed spheroids is provided as Supplementary Information (Online Resource 1).

### Ishikawa cells form polarized spheroids in 3D cell culture

**Fig. 1:**
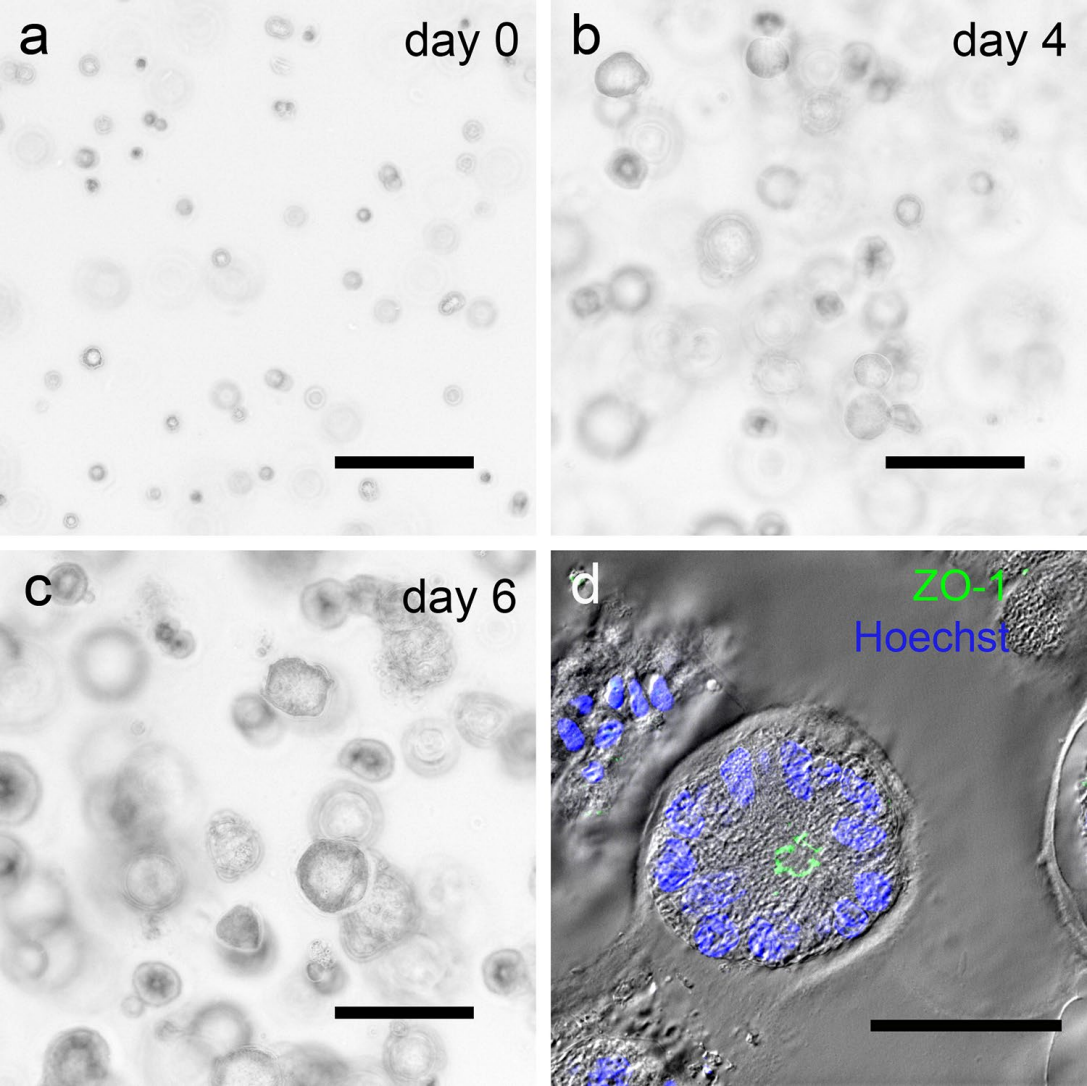
Ishikawa spheroids in cell culture and morphological confirmation of cell polarity. **a**–**c** show the development of Ishikawa spheroids in cell culture from day 0 to day 6. Microscopic phase contrast images were inverted. Scale bars: 150 µm. **d** shows a representative polarized Ishikawa spheroid on day 6. Nuclei are stained with Hoechst 33,342 (blue), tight junctions with anti-ZO-1 antibody (green). Scale bar: 30 μm

The growth of Ishikawa spheroids in cell culture was documented from day 0 to day 6 as shown in Fig. 1. On day 0, single cells were observed in Matrigel™ drops shortly after preparation. On day 4, the first small spheroids were formed, which were then treated with E2, P4, MPA or hCG. On day 6, the Ishikawa spheroids began to form a lumen and grew to a size of 30–250 µm by day 8. For further evaluation day 6 and 8 spheroids were used. The diameter of these spheroids was in the range of 50 µm. The nuclei of the epithelial cells were located on the basal side and the tight junction marker ZO-1 could be detected at the apical side of the lateral membrane demonstrating that the epithelium had polarized towards the lumen of the spheroid (Fig. 1d).

### Gestagens reduce proliferation of Ishikawa spheroids

**Fig. 2:**
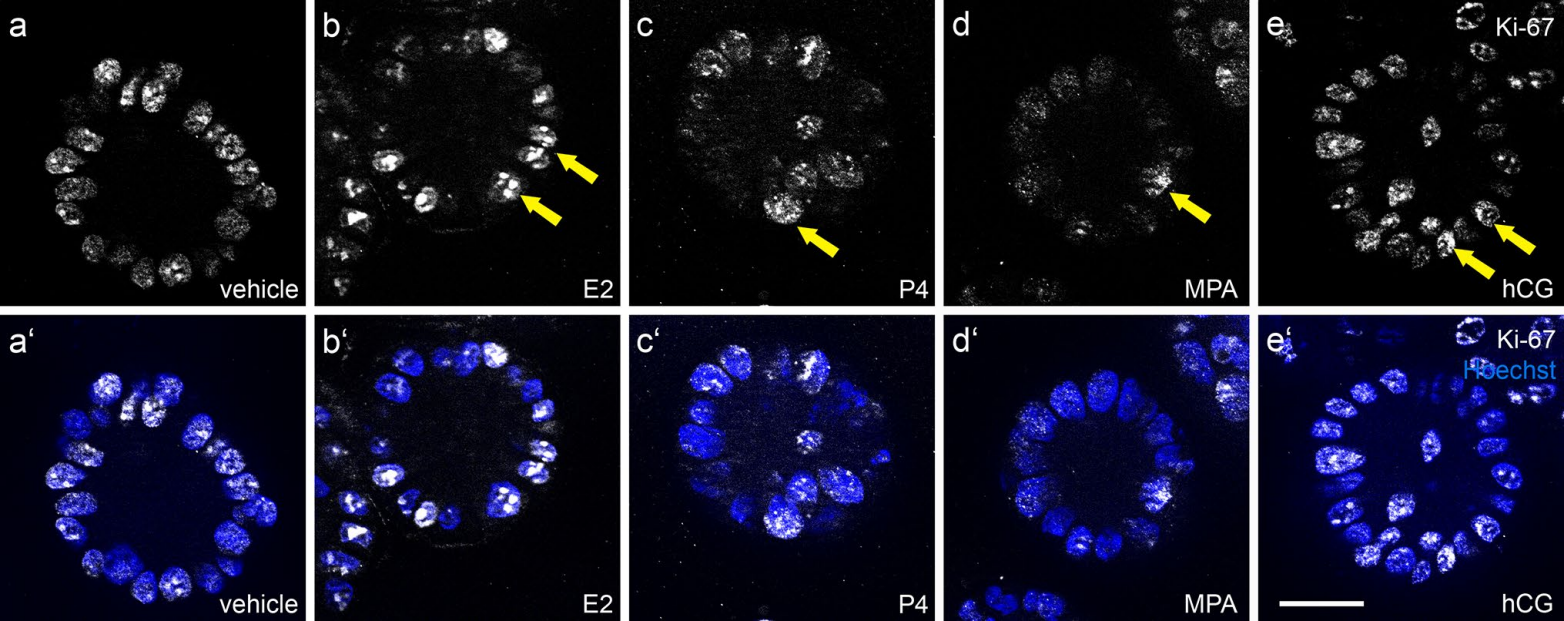
Influence of ovarian steroid hormones and hCG on Ishikawa spheroid proliferation. Images show Ishikawa spheroids stained for the proliferation marker Ki-67 after 4 days of stimulation with estradiol/E2 (**b/b’**), progesterone/P4 (**c/c’**), medroxyprogesterone acetate/MPA (**d/d’**) or human choriogonadotropin/hCG (**e/e’**). **a/a’** show the control without hormones. Arrows highlight the Ki-67 positive nuclei. Grey-scale pictures for Ki-67 in (**a**–**e)** and combination with nuclear staining (Hoechst; blue) in (**a**’–**e**’). Scale bar: 20 µm

To find out whether steroid hormones and hCG have an impact on proliferation of Ishikawa cells, we stained spheroids with antibodies detecting the proliferation marker Ki-67 (Fig. 2) after 4 days of stimulation. Proliferation of individual cells is characterized by a positive signal of the nuclei. A reduction of the Ki-67 signal was observed in the presence of progesterone (c/c’) and MPA (d/d’), whereas a higher number of nuclei were stained in the control (a/a’) and in the presence of either E2 (b/b’) or hCG (e/e’) stimulation. All experiments were performed three times with identical results. Together, the observations show that Ishikawa cells are sensitive to steroids and their proliferation is inhibited by gestagen treatment.

### Gestagens and hCG induce the distribution of the desmosomal plaque protein desmoplakin

**Fig. 3:**
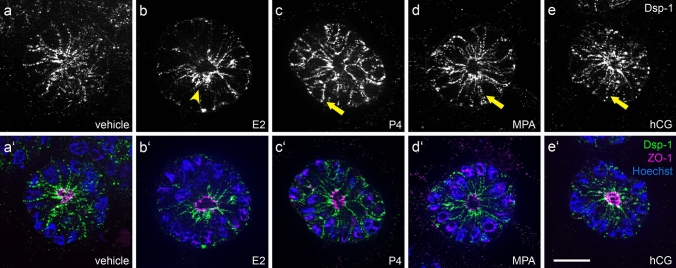
Influence of ovarian steroid hormones and hCG on localization of desmoplakin 1. Images show Ishikawa spheroids after 4 days of stimulation with estradiol/E2 (**b/b’**), progesterone/P4 (**c/c’**), medroxyprogesterone acetate/MPA (**d/d’**) or human choriogonadotropin/hCG (**e/e’**). **a/a’** show the control without hormones. Arrowhead highlights subapical accumulation of Dsp-1 expression (**b**). Arrows highlight Dsp-1 redistribution to the basolateral membrane (**c** and **d**). Grey-scale pictures for Dsp-1 (**a**–**e**) or in green combined with tight junctional staining (ZO-1, magenta) and Hoechst (blue) in (**a**’–**e**’). Scale bar: 20 µm

To investigate the effect of steroid hormones and hCG on the desmosomal plaque protein desmoplakin 1 (Dsp-1), Ishikawa spheroids were treated with the hormones starting on day 4 of cell culture. Experiments were performed three times with equal results. After 4 days of stimulation with E2, spheroids showed a subapical accumulation of Dsp-1 signal with sparse basolateral localization (Fig. 3b, b’). Stimulation with progesterone and MPA (Fig. 3c/c’, d/d’) induced an increased basolateral distribution of Dsp-1 with reduced apical enrichment. ZO-1 remained at the apical side of the lateral membrane next to the lumen in all instances.

### α6-integrin but not β4-integrin redistribute in response to gestagen and hCG stimulation

**Fig. 4:**
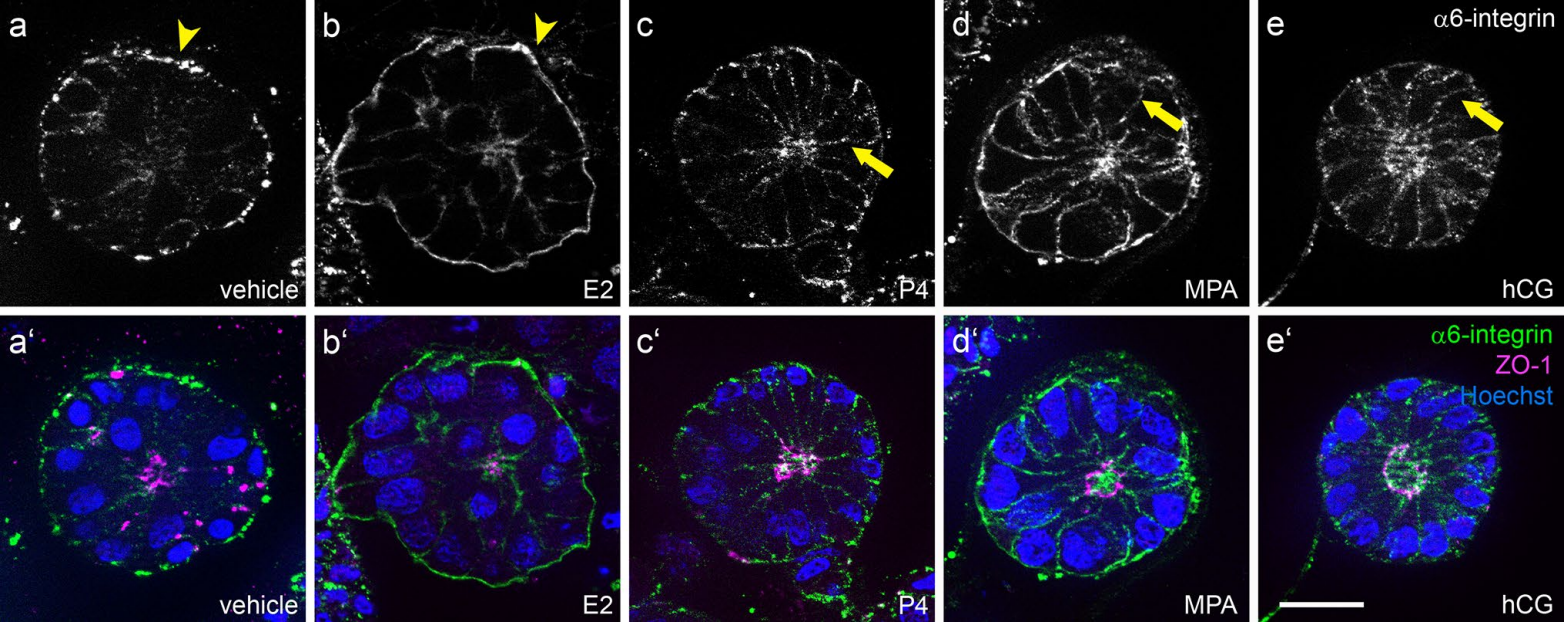
Influence of ovarian steroid hormones and hCG on localization of α6-integrin. Images show Ishikawa spheroids after 2 days of stimulation with E2/estradiol (**b/b’**), P4/progesterone (**c/c’**), medroxyprogesterone acetate/MPA (**d/d’**) or human choriogonadotropin/hCG (**e/e’**). **a/a’** depict the control without hormones. Arrowheads highlight the basal localization of α6-integrin in (**a**, **b**). Arrows highlight the lateralization of the α6-integrin signal in (**c**–**e**). Grey-scale pictures for α6-integrin (**a**–**e**) or in green combined with tight junctional staining (ZO-1, magenta) and Hoechst (blue) in (**a**’–**e**’). Scale bar: 20 µm

**Fig. 5:**
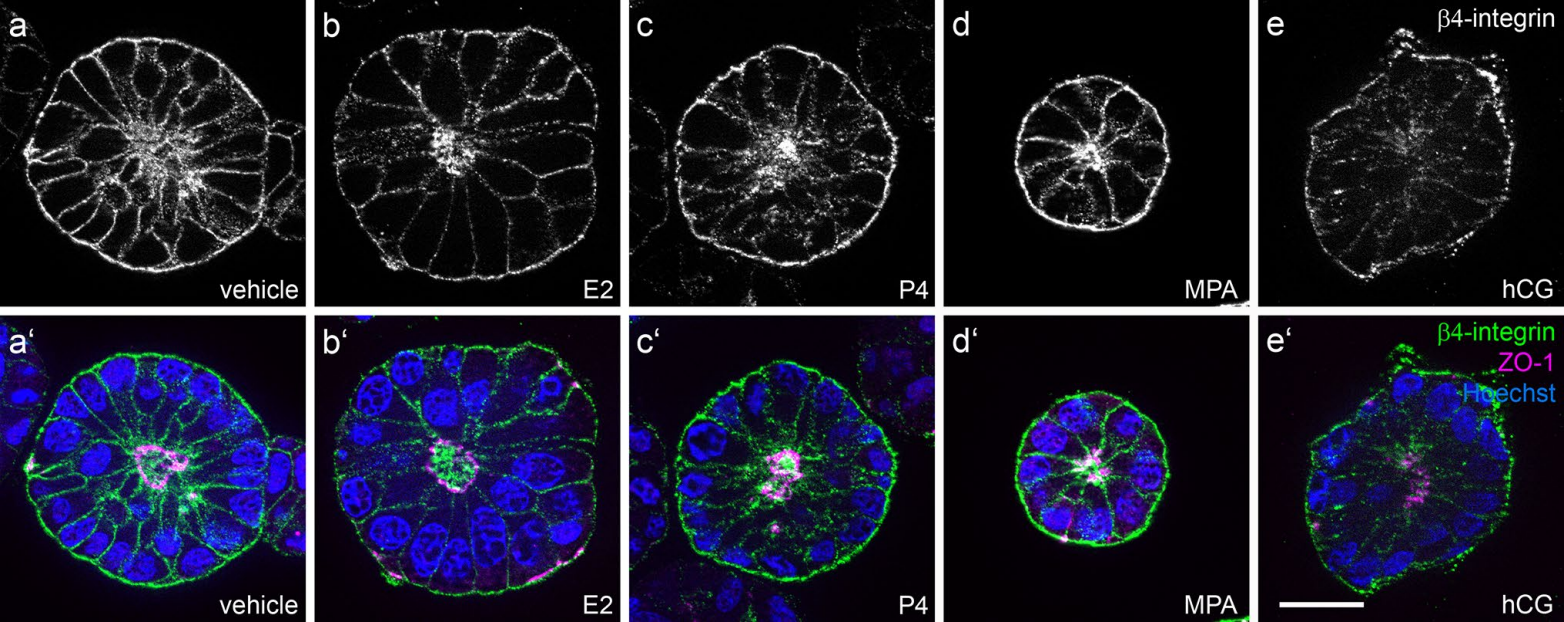
Influence of ovarian steroid hormones and hCG on localization of β4-integrin. Images show Ishikawa spheroids after 2 days of stimulation with E2/estradiol (**b/b’**), P4/progesterone (**c/c’**), medroxyprogesterone acetate/MPA (**d/d’**) or human choriogonadotropin/hCG (**e/e’**). **a/a’** represent the control without any hormones. Grey-scale pictures for β4-integrin (**a**–**e**) or in green combined with tight junctional staining (ZO-1, magenta) and Hoechst (blue) in (**a**’–**e**’). Scale bar: 20 µm

Ishikawa spheroids presented a predominantly basal signal for α6-integrin in the control and after 2 days of E2 stimulation (Fig. 4a/a’, b/b’). Stimulation with either progesterone, MPA or hCG induced an extension of the α6-integrin signal to the lateral cell borders (Fig. 4c–e, c’–e’). In contrast, β4-integrin staining of Ishikawa spheroids showed an equal basolateral distribution at all conditions (Fig. 5a–e, a’–e’).

### α6-integrin distribution is affected by the menstrual cycle stage in biopsies of ART patients

Since only α6-integrin localization changed from basal to lateral membrane staining in Ishikawa spheroids under P, MPA and hCG treatment, we examined only this marker in human primary tissue. Biopsies were obtained from 11 women undergoing ART.

**Fig. 6:**
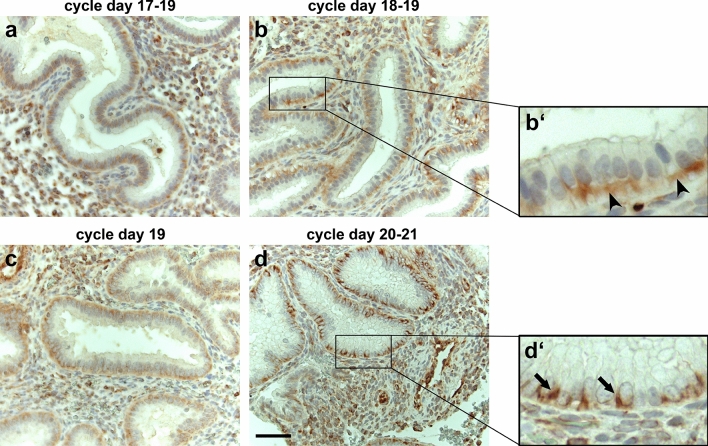
α6-Integrin staining during days 17–21 of the menstrual cycle of ART patients. Images show the expression pattern of α6-integrin during the menstrual cycle between days 17 and 21 in biopsies of women who underwent an ART cycle. **a** (menstrual cycle days 17–19), **b/b’** (days 18–19) and **c** (day 19) represent non-receptive biopsies before the WOI. **d/d’** (days 20–21) represent a potential receptive state during the WOI. Arrowheads depict a basal localization of α6-integrin (**b’**), while arrows highlight more laterally localized α6-integrin positive membrane staining (**d’**). Chromogen: AEC. Nuclear staining: Hematoxylin. Scale bar: 50 µm

Figure 6 illustrates the menstrual cycle-dependent expression of α6-integrin in four representative samples assigned to days 17–21 according to the Noyes criteria (Noyes et al. 1950). Although all biopsies were taken between 137 and 150 h after the first progesterone administration (corresponding to ~ day 20), a closer look at the histology of the biopsies revealed that their cycle-dependent differentiation status differed considerably. We used a modified dating method with parameters including Noyes criteria and immunohistochemical detection of estrogen and progesterone receptors and the proliferation marker Ki-67 as described in more detail by Alfer et al. (2020). The cause of this inhomogeneity is still unknown. However, similar deviations also occur in fertile patients who have not undergone intervention (Coutifaris et al. 2004; Lenton et al. 1984; Lindhard et al. 2006). α6-integrin was mainly detected on the basal aspect of endometrial epithelial cells between days 17 and 19 (Fig. 6a, b/b’, c). A much more lateral staining of α6-integrin, however, became discernible on days 20 and 21 (Fig. 6d/d’).

## Discussion

During the window of implantation and in preparation for embryo implantation, a substantial remodeling of the human endometrium takes place. In previous studies, we observed a redistribution of desmosomal and adherens junctions in human endometrial epithelial cells during the progesterone-dominated luteal phase of the menstrual cycle (Buck et al. 2012) and in a 3D culture system with three differently polarized cell lines (Buck et al. 2015). In this study, we could show a redistribution of the desmosomal plaque protein desmoplakin 1 in Ishikawa cells after stimulation with progesterone, medroxyprogesterone acetate and hCG in vitro. We further found that the extracellular matrix adhesion receptor α6-integrin, which has been localized to hemidesmosomes (Jones et al. 1991; Nievers et al. 1999; Stepp et al. 1990), also redistributed in response to hormonal stimulation from the basal plasma membrane to the basolateral plasma membrane. In contrast, the hemidesmosome-specific β4-integrin did not show such a redistribution.

### Effect of steroid hormones and hCG on polarized endometrial spheroids

The steroid hormones 17β-estradiol, progesterone and medroxyprogesterone acetate were applied to polarized, lumen-containing Ishikawa EECs to simulate the in vivo situation during the menstrual cycle.

The decision to include also the pregnancy hormone hCG as a supplement was based on the results obtained by intrauterine application of hCG prior to embryo transfer in clinical settings (Bielfeld et al. 2019). Liu et al. (2019) showed an improvement in implantation rate, pregnancy rate and live birth rate with intrauterine administration of hCG 3 days prior to cryo transfer. For the in vivo studies, 100–1000 IU hCG were used (Craciunas et al. 2016; Mansour et al. 2011; Strug et al. 2016). We extrapolated from these values an hCG concentration of 50 IU per ml cell culture medium for the in vitro experiments.

The proliferation of the spheroids was detected with the proliferation marker Ki-67 (Gerdes et al. 1984). Our results confirmed the basic assumption that the moderately differentiated Ishikawa cell line with verified steroid hormone receptors (Lessey et al. 1996; Nishida 2002) can react adequately to the steroid hormones by showing a physiological reaction. We could confirm that the proliferation was inhibited by progesterone and MPA in comparison to the treatment with E2 and hCG or the vehicle.

The luteinizing hormone/choriogonadotropin receptor (LHCGR) has been detected on primary human endometrial epithelial cells and found to be functional (Sacchi et al. 2018). Furthermore, the expression and production of hCG have been detected in human secretory endometrium (Zimmermann et al. 2012). In Ishikawa cells, the LHCGR has been identified but stimulation with hCG did not lead to the expected increase in intracellular cAMP (Viswanath et al. 2007). Srisuparp et al. (2003) showed hCG stimulation activated the MAPK pathway instead of cAMP in primary baboon endometrial cells. Due to these inconsistent findings, it is not clear which signaling pathway is activated by hCG in Ishikawa cells but our results show that hCG has effects on proliferation and junctional remodeling.

## Lateral cell–cell adhesion via desmosomes

Lateral cell–cell contacts that provide mechanical strength to simple polarized epithelia consist of a tripartite complex (Farquhar and Palade 1963). Besides the most apically localized zonulae occludentes, or tight junctions, it includes the actin-associated adherens junctions (zonulae adhaerentes) and the keratin filament network-anchoring desmosomes (maculae adhaerentes). Staining against the tight junction protein ZO-1 was used to visualize lumen formation inside the Ishikawa cell spheroids and provide evidence for EEC apico-basal polarization. Visible changes in the lateral distribution of tight junctions were not expected here, since cyclic changes in EEC tight junctions can only be detected by ultrastructural assessment (Murphy et al. 1982, 1992). To prove our concept that a redistribution of adherens junction markers reflects changes in epithelial cell polarity, we used the plaque protein desmoplakin 1 as a marker for desmosomal cell–cell adhesion. The same rationale was used in other studies investigating the involvement of desmosomes in implantation in different species (Illingworth et al. 2000; Preston et al. 2004, 2006). These studies showed that either the localization of epithelial junctions or their expression rate is changed during the phase that is referred to as the WOI in humans.

### Transmembrane cell–matrix adhesion proteins: α6- and β4-integrin

Beside the lateral junctions, we also studied the distribution of the basally localized α6- and β4-integrins as a function of hormonal control. Integrins are heterodimeric integral membrane proteins with an alpha and a beta chain. In mammals, 18 different alpha and 8 different beta subunits can assemble into 24 different heterodimers (Barczyk et al. 2010; Takada et al. 2007). Their main function is cell-extracellular matrix adhesion and signaling. α6β4-integrin (ITGA6:ITGB4) is a receptor for laminin 5 (now classified as laminin 3-3-2) in epithelial cells, which is found in hemidesmosomes and is connected to intermediate filaments (Nievers et al. 1999). The alpha 6 chain subunit can associate with beta 1 or with beta 4 subunit (Mercurio et al. 2001). Expression of α6- and β4-integrin in human endometrium has first been described by Lessey et al. (1992) and Tabibzadeh (1992) where it localized to the basolateral surface of endometrial glandular cells. Studies of the distribution of integrins in endometrial cancer (Lessey et al. 1995) revealed that α6-integrin staining becomes more diffuse and no longer spatially restricted to the basal portion of cells. In general, alterations in the appearance of laminin-receptors such as α6β4-integrin are often seen in malignant epithelial tumors. Also, in breast cancer cells the expression of both chains is either redistributed over the entire cell surface or significantly reduced. This altered pattern of expression is paralleled by a lack of detection of basement membrane laminin and collagen type IV (Natali et al. 1992).

Studies on the menstrual cycle (Albers et al. 1995) have shown that α6-integrin newly appears in the lateral membranes of epithelial cells during the secretory phase of the menstrual cycle. The authors proposed that the redistribution of α6-integrin subunits to lateral membranes initiates the formation of α6β1-complexes, since β4-integrin subunits were not found in the lateral membrane. These changes may reflect changes in the apico-basal polarization of the epithelial cells in preparation for trophoblast penetration both from the apical and basal side (Denker 1993). The results of Albers et al. (1995) are in accordance with our studies. In the WOI biopsies of patients undergoing ART, we observed a more lateral membrane staining of α6-integrin in the progesterone-dominated mid-secretory phase of the menstrual cycle. In addition, we were able to demonstrate more lateralization of the α6-integrin signal under stimulation with P4, MPA and hCG in the Ishikawa spheroids.

Although Murray et al. (1999) claimed no correlation of the expression of α6- and β4-integrin with uterine receptivity they described a diffuse immunostaining of α6 subunit around the lateral cell membranes during the mid-secretory phase whereas staining for the beta 4 subunit was intense only at the base of glandular cells. Because the staining patterns for α6- and β4-integrin differ, they also suggested that both α6β1 and α6β4 participate in epithelial anchorage.

Finally, Tanaka et al. (2009) could show that the remodeling of human endometrial epithelium is regulated by extracellular matrix, i.e., laminin. Expression of laminin is reduced in the progesterone-dominated mid- to late secretory phase compared to the estrogen-dominated proliferative phase.

## Conclusion

In the 3D in vitro system of gland-like Ishikawa spheroids, we could show that hormonal stimulation can induce a redistribution of adhesion proteins (summarized as Supplementary Information: Online Resource 2). This suggests that the 3D Ishikawa model system might be a useful tool to mimic the conditions during the window of implantation. The current results, in conjunction with previous studies, provide further credibility for the idea that a decrease in polarity is linked to EEC receptivity. This junction-based concept of polarity provides a simple mechanical mechanism for supporting and facilitating embryo invasion and implantation. The results obtained are therefore of potential relevance for diagnostic assessment and therapeutic success predictions of patients undergoing ART treatment.

## Supplementary Information

Below is the link to the electronic supplementary material.

Supplementary file1 (PDF 432 KB)

Supplementary file2 (PDF 416 KB)
