# Supplementary material for: Steroid hormones and human choriogonadotropin influence the distribution of alpha6-integrin and desmoplakin 1 in gland-like endometrial epithelial spheroids
Source: Histochem Cell Biol. 2021 Jan 27;155(5):581–91. doi: 10.1007/s00418-020-01960-z (PMC8134296; doi:10.1007/s00418-020-01960-z)
Supplement: Supplementary file 1 — Supplementary file1 (PDF 432 KB) [file 418_2020_1960_MOESM1_ESM.pdf]

Online Resource 1: Number of experiments and assessed spheroids

IHC antibody	condition	1st experiment [number of assessed spheroids]	2nd experiment [number of assessed spheroids]	3rd experiment [number of assessed spheroids]	in total [number of assessed spheroids]	spheroids showing the described IHC patterns [total number]	[%]
**Ki-67**	vehicle	6	5	7	18	13	72,22
	E2	6	5	14	25	20	80,00
	P4	4	5	9	18	14	77,78
	MPA	4	5	10	19	16	84,21
	hCG	6	5	12	23	16	69,57
**desmoplakin 1**	vehicle	9	11	16	36	32	88,89
	E2	6	6	9	21	20	95,24
	P4	5	4	7	16	11	68,75
	MPA	7	7	11	25	22	88,00
	hCG	4	5	11	20	18	90,00
**α6-integrin**	vehicle	4	18	12	34	28	82,35
	E2	5	10	5	20	16	80,00
	P4	4	17	7	28	22	78,57
	MPA	5	10	7	22	19	86,36
	hCG	4	13	8	25	22	88,00
**β4-integrin**	vehicle	16	5	11	32	28	87,50
	E2	15	7	9	31	24	77,42
	P4	10	4	10	24	22	91,67
	MPA	16	10	9	35	23	65,71
	hCG	11	6	9	26	23	88,46

Abbreviations: IHC = immunohistochemistry; E2 = 17β-estradiol; P4 = progesterone; MPA = medroxyprogesterone acetate; hCG = human choriogonadotropin.
