# Supplementary material for: Steroid hormones and human choriogonadotropin influence the distribution of alpha6-integrin and desmoplakin 1 in gland-like endometrial epithelial spheroids
Source: Histochem Cell Biol. 2021 Jan 27;155(5):581–91. doi: 10.1007/s00418-020-01960-z (PMC8134296; doi:10.1007/s00418-020-01960-z)
Supplement: Supplementary file 2 — Supplementary file2 (PDF 416 KB) [file 418_2020_1960_MOESM2_ESM.pdf]

Online Resource 2: Summary of changes in hormonally stimulated Ishikawa cell spheroids

IHC antibody	condition	nuclear staining	subapically concentrated lateral staining	equally distributed lateral staining	basal membrane staining
**Ki-67**	vehicle	++	-	-	-
	E2	+++	-	-	-
	P4	+	-	-	-
	MPA	+	-	-	-
	hCG	++	-	-	-
**desmoplakin 1**	vehicle	-	+	+	-
	E2	-	++	-	-
	P4	-	-	++	-
	MPA	-	-	++	-
	hCG	-	+	++	-
**α6-integrin**	vehicle	-	-	-	++
	E2	-	-	-	++
	P4	-	-	++	+
	MPA	-	-	++	+
	hCG	-	-	++	+
**β4-integrin**	vehicle	-	-	+	++
	E2	-	-	+	++
	P4	-	-	+	++
	MPA	-	-	+	++
	hCG	-	-	+	++

Strength of staining and main localization was classified to: “**+**” low, “**++**” middle and “**+++**” strong. “-“ means “no staining”.

Abbreviations: IHC = immunohistochemistry; E2 = 17β-estradiol; P4 = progesterone; MPA = medroxyprogesterone acetate; hCG = human choriogonadotropin.
